# MammoViT: A Custom Vision Transformer Architecture for Accurate BIRADS Classification in Mammogram Analysis

**DOI:** 10.3390/diagnostics15030285

**Published:** 2025-01-25

**Authors:** Abdullah G. M. Al Mansour, Faisal Alshomrani, Abdullah Alfahaid, Abdulaziz T. M. Almutairi

**Affiliations:** 1 Radiology and Medical Imaging Department, College of Applied Medical Sciences, Prince Sattam Bin Abdulaziz University, Alkharj 11942, Saudi Arabia; 2 Department of Diagnostic Radiology Technology, College of Applied Medical Science, Taibah University, Medinah 42353, Saudi Arabia; 3 College of Computer Science and Engineering, Taibah University, Yanbu 46421, Saudi Arabia; 4 Department of Computer, College of Science and Humanities, Shaqra University, Shaqra 11961, Saudi Arabia

**Keywords:** BIRADS classification, deep learning, ResNet50, Vision Transformer, SMOTE, mammogram analysis, medical imaging, breast cancer detection

## Abstract

**Background:** Breast cancer screening through mammography interpretation is crucial for early detection and improved patient outcomes. However, the manual classification of mammograms using the BIRADS (Breast Imaging-Reporting and Data System) remains challenging due to subtle imaging features, inter-reader variability, and increasing radiologist workload. Traditional computer-aided detection systems often struggle with complex feature extraction and contextual understanding of mammographic abnormalities. To address these limitations, this study proposes MammoViT, a novel hybrid deep learning framework that leverages both ResNet50’s hierarchical feature extraction capabilities and Vision Transformer’s ability to capture long-range dependencies in images. **Methods:** We implemented a multi-stage approach utilizing a pre-trained ResNet50 model for initial feature extraction from mammogram images. To address the significant class imbalance in our four-class BIRADS dataset, we applied SMOTE (Synthetic Minority Over-sampling Technique) to generate synthetic samples for minority classes. The extracted feature arrays were transformed into non-overlapping patches with positional encodings for Vision Transformer processing. The Vision Transformer employs multi-head self-attention mechanisms to capture both local and global relationships between image patches, with each attention head learning different aspects of spatial dependencies. The model was optimized using Keras Tuner and trained using 5-fold cross-validation with early stopping to prevent overfitting. **Results:** MammoViT achieved 97.4% accuracy in classifying mammogram images across different BIRADS categories. The model’s effectiveness was validated through comprehensive evaluation metrics, including a classification report, confusion matrix, probability distribution, and comparison with existing studies. **Conclusions:** MammoViT effectively combines ResNet50 and Vision Transformer architectures while addressing the challenge of imbalanced medical imaging datasets. The high accuracy and robust performance demonstrate its potential as a reliable tool for supporting clinical decision-making in breast cancer screening.

## 1. Introduction

Breast cancer (BC) is known as one of the most common women-affected malignancies, with 12.5% new reported cases worldwide as of 2020 [1]. The tumor is formed when the cells in the breast grow uncontrollably (can be felt as a lump). These cells can spread through metastasis to other parts of the body by invading surrounding tissues [2]. Women over 40 years of age, specifically in their 50s or 60s, have a higher chance of developing a breast tumor [3]. BC is characterized by hormone receptor status (ER, PR, and HER2/neu overexpression), which is known as a significant molecular subtype with biological and clinical features [4]. The most common symptoms of BC include pain in the breast, lump in the breast or underarm area, skin dimpling, changes in breast shape or size, and nibble discharge followed by severe pain. Genetic mutations, including BRCA1 and BRCA2, family history, hormonal factors, environmental exposures, and lifestyle choices are included in breast cancer risk factors [5].

Traditionally, the diagnosis and classification of breast cancer relied heavily on ultrasound, mammography, MRI, and histopathological examination [6]. Mammography is a widely used screening tool that helps in the early-stage detection of BC by creating detailed images of the breast using low-dose X-rays [7]. This approach has been effective in the identification of calcifications and other abnormalities in the breast tissues. However, it has limitations, such as reduced sensitivity in dense breast tissue and false positives [8]. Ultrasound, often used as an adjunct to mammography, produces images of the breast using high-frequency sound waves. This technique effectively differentiates between fluid-filled cysts and solid masses, which ultimately helps guide needle biopsies. However, this method is operator-dependent and less effective at detecting small tumors [9].

Histopathology, the gold standard for diagnosing BC, involves the surgical removal of a tissue sample from the breast (biopsy). This sample is then stained and examined by a pathologist to detect cancerous cells and determine the type and stage of the cancer. Although histopathology provides a definitive diagnosis and detailed information on cancer, it can be uncomfortable and carries risks such as infection and bleeding, as it is an invasive procedure. Additionally, it is a costly and time-consuming approach [10]. Magnetic Resonance Imaging (MRI) is a powerful diagnostic tool used in the traditional detection and characterization of breast cancer [11]. Unlike mammography and ultrasound, which rely on X-rays and sound waves, the MRI produces detailed breast tissue scans and images using strong magnetic fields and radio waves. This modality is particularly valuable for its high contrast resolution, which can distinguish between different types of tissues and detect abnormalities that might not be visible with other imaging techniques. It is susceptible, gives a detailed analysis, and is effective in screening high-risk patients. However, this approach can give false positives and requires special training and expertise to interpret the results [12].

Recent advances in machine learning (ML) and deep learning (DL) have been introduced to overcome these limitations and develop automated approaches for BC detection and classification. The SVM algorithm (Support Vector Machine) classified breast lesions based on the extracted features from MRI scans [13]. This method provided high accuracy and efficiency in detecting and distinguishing malignant and benign cases. However, this approach usually struggles with larger datasets and high-dimensional spaces, which could lead to excessive time consumption and overfitting if not tuned properly. The Naïve Bayes approach applied Bayes’ theorem to classify BC by assuming independence among the extracted features. This simplified the modeling of BC diagnosis. However, this assumption often does not hold, potentially leading to suboptimal performance [14]. The RF model (known as Random Forest), an ensemble of decision trees, classifies MRI-extracted features. This method effectively handles high-dimensional data and also improves diagnostic performance. However, this approach is usually less interpretable than the other methods, which makes it challenging to understand the contribution of individual features to the prediction [15].

K-NN (the famous K-Nearest Neighbor method) detects breast lesions and provides straightforward classification by measuring the proximity of extracted features to training samples in feature space. Its performance is susceptible to the choice of distance metric and requires the entire dataset to be stored in memory, which can be inefficient for large datasets [16]. To overcome these challenges, some advanced DL approaches and architectures have been used in the early diagnosis and classification of BC. GANs (Generative Adversarial Networks) work by generating synthetic MRI images to augment training datasets. In this way, the method enhances the model’s robustness and improves diagnostic accuracy [17]. The Transfer Learning method combines and fine-tunes pre-trained models on BC MRI datasets, effectively extracting the existing knowledge to improve classification performance [18]. U-Net is another method designed specifically for biomedical image segmentation tasks. This enables precise delineation of cancer/tumor boundaries in the MRI scans for a better treatment strategy [19].

Deep Belief Networks (DBNs) contribute to improved classification performance in BC detection by learning complex representations of the MRI scan data [20]. RNNs (known as Recurrent Neural Networks) capture the temporal changes in tumor characteristics by analyzing the sequence of MRI scans over time. This approach gives enhanced diagnostic insights [21]. CNNs (Convolutional Neural Networks) achieved high diagnostic accuracy in the detection and classification of BC as they could automatically learn the spatial hierarchies of the extracted features from MRI scans [22].

Despite significant advancements in computer-aided detection (CAD) systems, the automated classification of mammograms continues to face several challenges. One major issue is the difficulty in extracting complex imaging features that distinguish subtle abnormalities, as overlapping characteristics across BIRADS categories can lead to reduced classification accuracy. Additionally, class imbalance in medical imaging datasets, where certain BIRADS categories are severely underrepresented, poses a critical challenge. This imbalance often biases model predictions toward majority classes, thereby compromising the reliability of CAD systems in diagnostic applications. Furthermore, traditional CNNs are constrained by their localized receptive fields, limiting their ability to capture long-range spatial dependencies in mammograms. This shortcoming can result in a lack of contextual understanding, which is essential for accurate classification. These challenges hinder the performance of CAD systems in real-world clinical settings, highlighting the need for novel approaches that can overcome these limitations and enhance the reliability of automated mammogram classification. Therefore, the key contributions of this research are as follows:

- Feature Extraction with ResNet50: Employed a pre-trained ResNet50 model to extract robust and discriminative features from the imbalanced BIRADS dataset.
- Vision Transformer Model: Leveraged the power of the Vision Transformer architecture, a state-of-the-art deep learning model, to effectively extract and learn hierarchical representations from mammogram images.
- Hyperparameter Optimization: Utilized Keras Tuner to systematically explore and optimize hyperparameters of the Vision Transformer model, leading to improved performance and generalization.
- Model Evaluation: Conducted a rigorous evaluation of the proposed model on the balanced BIRADS dataset, demonstrating its effectiveness in accurately classifying mammogram images into their respective BIRADS categories.

The following sections are structured as follows: Section 2 reviews relevant prior work, Section 3 details the experimental methodology, Section 4 presents the experimental results, and Section 5 concludes this paper and discusses future research directions.

## 2. Related Work

In recent years, significant advancements have been made in the development of deep learning models for medical image analysis. These efforts can be broadly categorized into CNNs, transformer-based architectures, hybrid models, and specialized architectures such as U-Net and its variants. Below, we provide an overview of these categories and their application to mammogram classification and related tasks.

In [23], the authors proposed and implemented a CAD framework (known as AI-based computer-aided diagnosis) named ETECADx. This framework combines the ViT encoder and ensemble transfer learning of CNNs to improve BC diagnosis. The utilized dataset was INbreast and the model performed very well, achieving a 98.58% accuracy score for binary classification and a 97.87% accuracy score for multi-class classification. However, one possible limitation of the study was the need for a more diverse and more extensive dataset to validate the model’s efficiency.

CNNs with Swin Transformers were combined to perform the breast ultrasound image segmentation task [24]. This hybrid approach employed several modules, including a pyramid structure, to enhance boundary detection and feature extraction. The model performed very well on the UDIAT dataset and achieved a DSC score of 0.8725. Despite its advancements, the model showed limited segmentation accuracy for unclear lesion boundary images.

In [25], a 3D RetinaNet-based CNN algorithm was employed for BC detection and classification by examining the Ultrafast TWIST DCE-MRI scans/images. This method resulted in a detection accuracy rate of 90% and a sensitivity of 95%. However, the approach faced certain limitations, notably the requirement for high-quality imaging and substantial computational resources. Another KFLI model (Knowledge-Driven Feature Learning and Integration) was implemented [26]. It used DCE-MRI (known as dynamic contrast-enhanced MRI) data and DWI (diffusion-weighted imaging) data. This model achieved an accuracy score of 85% in the efficient diagnosis of BC. However, this study faced challenges related to data integration and variability.

In a comparison study [27], the researchers implemented CNN models to compare them with human readers. The researchers evaluated which of the two could distinguish malignant breast lesions from benign ones utilizing DCE images. Among the models, InceptionResNetV2 gained popularity as it achieved an AUC score of 0.895. However, the performance was limited due to image quality dependency issues. In [28], the U-Net method combined with information bottlenecks and transformer (IB-TransNet) was utilized for BC detection and segmentation tasks using ultrasound images. This algorithm prevented overfitting by using bottlenecks to remove the redundant features. Feature maps (high and low resolution) were also fused. This model achieved a dice score of 81.05% for BC segmentation. In another study, the researchers developed a CAD architecture that employed ensemble learning (multiple CNN models combined: VGGNet, DenseNet, and ResNet). An image fusion process was used to enhance the BC classification. The model surprisingly achieved an accuracy score of 94.62% on the BUSI dataset. However, the ensemble technique is time-consuming and complex; it can be challenging to apply it in clinical settings [29].

A 3D-Deep Attentive Model (U-Net) was integrated with a transformer architecture for BC segmentation. The model processed volumetric data from ABVS (the Automated Breast Volume Scanners) and focused on the relevant features. This method was able to capture long-range dependencies and achieved a DSC score of 0.917. However, extensive data training is highly required in deep learning approaches [30]. An advanced method (TR-IMUnet) was proposed by modifying the U-Net model combining a transformer architecture and multi-scale CNN model for BC detection and segmentation. This study processed the DCE-MRI images for the task. The experimental data showed that the Dice score increased by 4.27% (0.92). However, this model needs to be applied to a more diverse dataset to test its reliability and performance [31].

The CrossViT Model (also known as the Cross Vision Transformer) approach was integrated with an enhanced version of the MGO algorithm (Growth Optimizer) for BC detection. This model captured both the local and global features by combining the strengths of the ViT model and CNN. Compared to other approaches, the CrossViT-MGO method achieved a 1.59% higher accuracy score on the INbreast dataset [32]. In [33], authors demonstrated the effectiveness of combining self-attention transformers with compact convolution transformers (CCTs) and tokenlearner (TVIT) models for breast cancer classification using mammography images. Their approach achieved high performance while reducing computational resources and decision time. Similarly, a study [34] by Nehad et al. proposed the AMAN method, which uses a deep learning model (Xception) for feature extraction followed by gradient boosting for classification. This method achieved impressive results, including 87% accuracy and an AUC of 95% for mammography classification. Similarly, our work extends the use of deep learning to BIRADS classification with a hybrid ResNet50 and Vision Transformer architecture, focusing on the challenges of class imbalance and contextual feature extraction.

## 3. Materials and Methods

### 3.1. Dataset Description

The KAU-BCMD dataset, obtained from the Sheikh Mohammed Hussein Al-Amoudi Center of Excellence in Breast Cancer at King Abdulaziz University, is employed in this study. This comprehensive dataset encompasses 1416 patient cases, comprising 5662 mammography images and 405 ultrasound images from 205 corresponding cases. The mammography images were captured in DICOM format from two angles for each breast, ensuring standardized medical imaging quality. Three experienced radiologists participated in the annotation and evaluation process.

The dataset, collected during 2019–2020, includes detailed region-of-interest (RoI) segmentation and bounding box annotations created using MATLAB ’s image labeller. The distribution of cases across BIRADS categories is as follows:

1. BIRADS 1: 1865 images and corresponding labels;
2. BIRADS 3: 387 images and corresponding labels;
3. BIRADS 4: 102 images and corresponding labels;
4. BIRADS 5: 24 images and corresponding labels.

It is notable that while BIRADS 2 cases represent 48% of the total dataset, they are not included in the labeled portion as they represent benign findings. The dataset is publicly available through the Kaggle platform.

#### 3.1.1. Data Loading and Preprocessing Architecture

**Figure 1:**
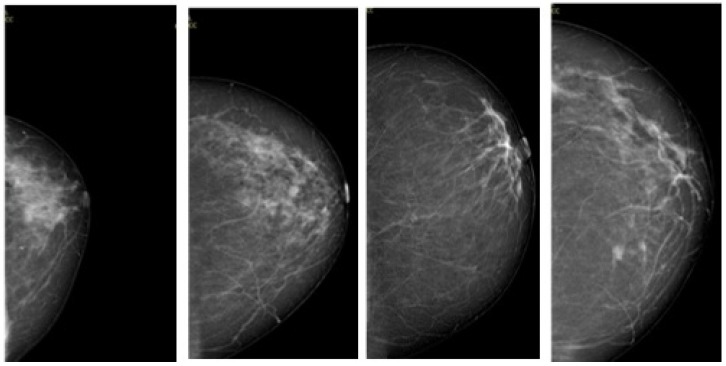
Visualizing the spectrum of breast density and architectural distortion across different BIRADS categories.

Our data processing pipeline employs TensorFlow’s I/O operations to handle the mammography images, which are standardized to three-channel RGB format and resized to uniform dimensions of 224 × 224 pixels. The pixel values undergo normalization using standardized preprocessing techniques. The dataset is organized using a pandas DataFrame structure with two primary columns containing file paths and corresponding BIRADS labels across four distinct categories (BIRADS 1, BIRADS 3, BIRADS 4, and BIRADS 5), with automated label assignment based on the directory structure. Data samples are shown in Figure 1. To ensure data integrity, comprehensive validation checks are performed, including shape verification, null value detection, and label encoding consistency verification.

#### 3.1.2. Feature Extraction Implementation

**Figure 2:**
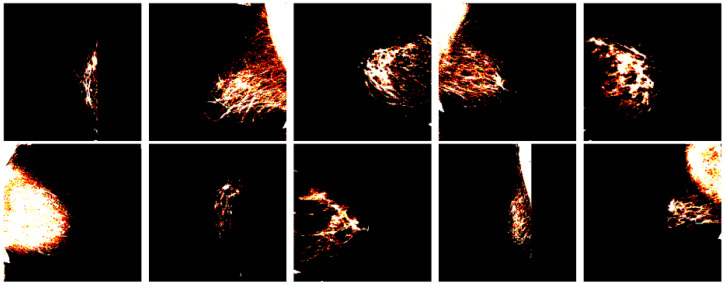
Sample Training Images from BIRADS Dataset.

The feature extraction process utilizes a modified ResNet50 architecture, pre-trained on ImageNet, with top layers excluded and input shape configured to (224, 224, 3). Global Average Pooling is applied to the extracted features. The dataset is partitioned using an 80:20 training–validation split ratio, with a fixed random state of 42 to ensure reproducibility. Stratified sampling maintains the original class distribution across both sets. Training samples are shown in Figure 2.

The choice of ResNet-50 as the backbone for the proposed MammoViT framework is motivated by its strong hierarchical feature extraction capabilities and proven effectiveness in medical image analysis. ResNet-50 introduces residual connections, which mitigate the vanishing gradient problem and allow for the training of deeper networks without loss of performance. This enables it to extract rich, multi-scale features that are crucial for accurately distinguishing subtle differences in mammographic images across BIRADS categories. Furthermore, ResNet-50 has been extensively validated on large-scale image datasets and has demonstrated robust performance in various transfer learning tasks, making it a reliable and efficient choice for feature extraction. By leveraging ResNet-50, we ensure that the input to the Vision Transformer is both informative and representative, enhancing the overall classification accuracy of the MammoViT architecture.

#### 3.1.3. Class Imbalance Management

To address class imbalance, we implemented SMOTE specifically on the training feature set, maintaining a random state of 42 for consistency and achieving balanced class distribution post-synthesis. Feature normalization is accomplished using StandardScaler, which is fit on the training data and subsequently applied to the validation set using the same parameters. This approach ensures robust preprocessing while maintaining data integrity and addressing the inherent class imbalance challenges in medical imaging datasets.

**Figure 3:**
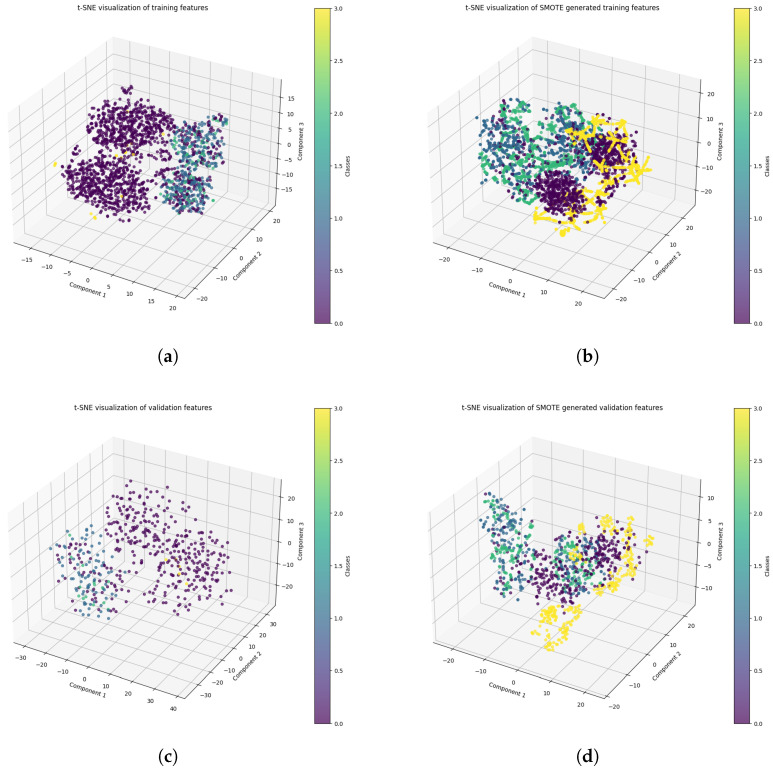
A t-SNE visualization of the original and SMOTE-balanced training and validation datasets. (**a**) Training features, (**b**) SMOTE training feature, (**c**) validation features, and (**d**) SMOTE validation feature.

Figure 3 presents the t-SNE visualization of feature distributions in both training and validation datasets before and after applying the SMOTE technique. The visualization comprises four subplots: (a) original training features, (b) SMOTE-generated training features, (c) original validation features, and (d) SMOTE-generated validation features. In the original feature space (Figure 3a,c), we can observe distinct clustering patterns but with notable class imbalance, which is particularly evident in the validation set. After applying SMOTE (Figure 3b,d), the feature distribution shows improved class balance and more uniform spatial distribution across all classes. This transformation demonstrates SMOTE’s effectiveness in addressing class imbalance while maintaining the inherent structure of the feature space. The color gradients from purple to yellow represent different class categories, with the three-dimensional representation capturing the complex relationships between features in our dataset. This visualization confirms that SMOTE not only balances the class distribution but also preserves the meaningful relationships between different classes in both training and validation sets.

### 3.2. Data Reshaping and Tensor Processing

Following the initial preprocessing and SMOTE balancing, the feature arrays undergo additional processing to ensure compatibility with deep learning architectures. The balanced dataset is first converted from TensorFlow tensors to NumPy arrays for intermediate processing. A secondary train–test split is performed on the SMOTE-balanced data, maintaining a 20% validation set with random state 42 for consistency.

The feature dimensionality is carefully managed through a reshape operation, with particular consideration for the feature space structure. The implementation handles 2048-dimensional feature vectors by reshaping them into a more suitable format for convolutional processing, specifically restructuring them into tensors with dimensions of (batch_size, 16, 16, 8). This transformation preserves the original feature information while adapting it to a format more conducive to deep learning operations.

To ensure data pipeline consistency, all processed arrays are converted back to TensorFlow tensors with appropriate data types—float32 for feature arrays and int32 for label arrays. The final tensor shapes are validated to maintain strict correspondence between features and their respective labels, providing a robust foundation for subsequent model training. This reshaping strategy effectively bridges the gap between the feature extraction phase and the neural network architecture while maintaining the integrity of the balanced dataset.

### 3.3. Proposed Architecture

**Figure 4:**
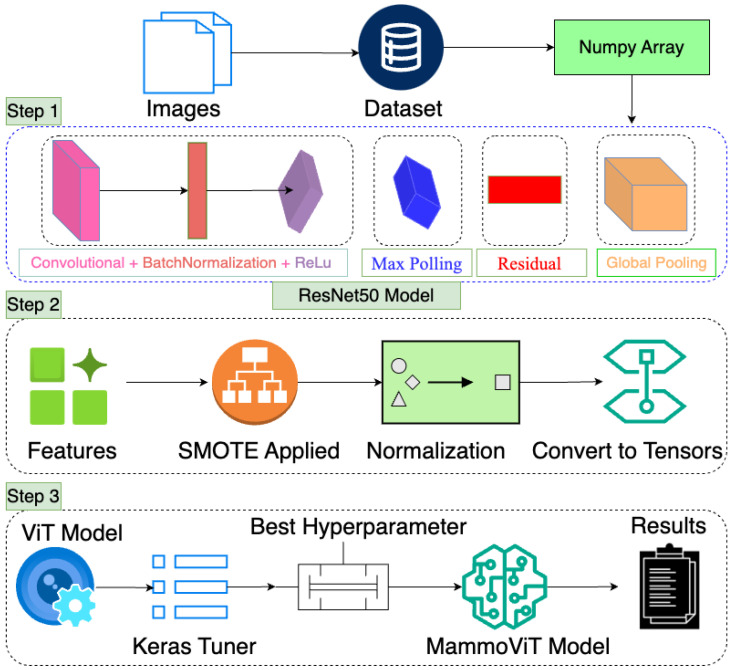
A step-by-step visualization of the MammoViT model, highlighting key elements.

The proposed MammoViT model is a deep learning approach for breast cancer detection, as shown in Figure 4. It involves three main steps: data preparation, feature extraction and augmentation, and model training and hyperparameter tuning. In the first step, images are preprocessed using a combination of convolutional layers, batch normalization, ReLU activation, max pooling, residual connections, and global pooling. The preprocessed images are then fed into a pre-trained ResNet50 model to extract features. The extracted features are augmented using SMOTE to address the class imbalance. The augmented features are normalized and converted into tensors. In the second step, the prepared tensors are fed into a ViT model for classification. The ViT model is optimized using Keras Tuner to find the best hyperparameters. The optimized ViT model is fine-tuned on the breast cancer dataset, resulting in the final MammoViT model. The MammoViT model demonstrates high accuracy in detecting breast cancer, making it a promising tool for early diagnosis and improved patient outcomes.

ViTs have gained significant popularity as they have brought remarkable advancement in the field of computer vision by applying transformer-based architecture to classification tasks. This process was initially designed for NLP (known as natural language processing) tasks, which handled image classification. CNNs extract local features through convolutional operations, limiting their ability to capture global context. In contrast, ViTs represent images as a sequence of patches and leverage self-attention to model long-range dependencies between these patches, leading to improved performance in tasks requiring global understanding.

At the core of ViTs is the process known as image patching, which generates non-overlapping image patches by dividing the given input images. For instance, an image of size 224 × 224 pixels can be divided into patches of size 16 × 16 pixels, resulting in 196 patches arranged in a 14 × 14 grid. Each non-overlapping patch (Ph) is transformed into a one-dimensional vector. Subsequently, a linear layer is employed to project these vectors into a higher-dimensional space. Mathematically, this projection can be expressed as Equation (1):

(1) $$\mathrm{Patch}\;\mathrm{Embedding}=M_{p}\cdot \mathrm{Ph}+B_{p}$$

where $M_{p}$ represents the weight matrix, and $B_{p}$ shows the bias. With the help of positional embeddings in the architecture, the positional information of these patch embeddings is retained. These are learnable vectors added to each patch embedding, ensuring that the spatial relationships between patches are preserved as they pass through the model.

These tokenized patches, combined with position-specific encoding information, are processed through a series of transformer encoder blocks. Each block’s architecture integrates two key elements as expressed in Equation (2): the multi-head self-attention (MHSA) mechanism that weighs patch relationships, and cascading feed-forward neural networks (FFNs). This structure allows the system to dynamically evaluate spatial correlations between patch elements.

(2) $$Attention\;\left(O,M,P\right)=softmax\left(OM^{T}/\sqrt{d_{k}}\right)P$$

where $d_{k}$ is the critical dimensions and (O, M, P) denote the linear projections of the input data.

The output is subsequently passed through a feed-forward network, which is applied to each position within the sequence. Equation (3) describes this process:

(3) $$\mathrm{FFN}\left(z\right)=\mathrm{ReLU}\left(T_{1}z+L_{1}\right)T_{2}+L_{2}$$

Here, $T_{1}$, $L_{1}$, $T_{2}$, and $L_{2}$ are learnable parameters that are unique to each encoder layer. In the ViT model, a unique classification token is prepended to the sequence of patch embeddings. After processing through all transformer encoder layers, the output corresponding to this classification token enters the section of fully connected layers. The final class probabilities are then obtained using a SoftMax function. This transformer-based approach in ViTs enables the model to capture more complex, challenging, and contextualized features from the input images, making it a powerful alternative to traditional CNNs in many vision-related tasks.

#### 3.3.1. Vision Transformer Model Design

Our proposed architecture implements a modified Vision Transformer (ViT) specifically designed for medical image classification. The model accepts input tensors of shape (16, 16, 8) and processes them through a series of carefully structured transformer blocks. The architecture begins with a patch embedding layer utilizing a convolutional approach, where patch sizes are dynamically optimized between 2 and 4 pixels. The embedding process projects these patches into a higher-dimensional space, with the projection dimension varying between 32 and 64 features.

The core of our architecture consists of multiple transformer blocks as shown in Table 1 (ranging from 2 to 5), each incorporating multi-head self-attention mechanisms. The number of attention heads in each transformer block is optimized between 2 and 8, allowing the model to capture different types of relationships in the feature space. Each transformer block includes the following:

- Multi-head self-attention layer;
- Layer normalization (epsilon = 1 × 10^−6^);
- Two dense layers with GELU activation;
- Dropout layers for regularization (rate optimized between 0.3 and 0.7);
- Residual connections maintained through layer normalization.

#### 3.3.2. Hyperparameter Optimization Strategy

We employed Keras Tuner’s RandomSearch algorithm for systematic hyperparameter optimization. The tuning process explores various configurations:

- Learning rates: 0.01, 0.001, 0.0001;
- Projection dimensions: 32, 48, 64;
- Transformer blocks: 2, 3, 4, 5;
- Attention heads: 2, 4, 6, 8;
- Dropout rates: 0.3 to 0.7 with 0.1 steps.

The optimization process encompasses ten trials, each evaluating different hyperparameter combinations against validation accuracy. The model is configured with the Adam optimizer and sparse categorical cross-entropy loss, trained over 50 epochs with a batch size of 256. To prevent overfitting, we implemented L1–L2 regularization (L1 = 1 × 10^−5^, L2 = 1 × 10^−4^) on the dense layers within the transformer blocks.

The classification head includes a global average pooling layer, followed by a dense layer with softmax activation, aligned with the number of target classes. The final model configuration is selected based on the optimal validation accuracy across all trials, ensuring robust performance on unseen data.

## 4. Results

In this study, MammoViT was implemented and evaluated using a comprehensive suite of deep learning and data analysis tools. The implementation was carried out using TensorFlow and Keras frameworks, explicitly utilizing the ResNet50 architecture as the backbone model. For data preprocessing and analysis, scientific computing libraries, including NumPy, Pandas, and Scikit learn, were used. The SMOTE algorithm was utilized to address class imbalance issues. The model’s performance was systematically evaluated using multiple metrics: loss and accuracy curves to track training dynamics; confusion matrices to visualize prediction patterns; classification reports detailing precision, recall, and F1-scores; and probability distribution graphs to assess prediction confidence.

### 4.1. Classification Report

Table 2 presents a classification report derived from the BIRADS dataset, where the model’s performance is evaluated across four BIRADS classes: 1, 3, 4, and 5. Each class label represents different diagnostic categories in the BIRADS system, commonly used for breast cancer risk assessment.

For each class, precision (*P*), recall (*R*), and F1-score (*F*) provide insights into the model’s accuracy in distinguishing between these specific categories: The model’s performance metrics reveal high precision (P), recall (R), and F1-scores across BIRADS classes, with a precision of 0.99 for BIRADS 1, a recall of 0.99 for BIRADS 3, and a perfect F1-score of 1.00 for BIRADS 5. The dataset comprises 1193 samples with varying class support, from 323 instances of BIRADS 1 to 311 instances of BIRADS 5. The model achieves an overall accuracy of 0.97, with both macro and weighted averages consistently at 0.97, indicating balanced and reliable classification performance across all BIRADS categories.

### 4.2. Accuracy and Loss Trends

**Figure 5:**
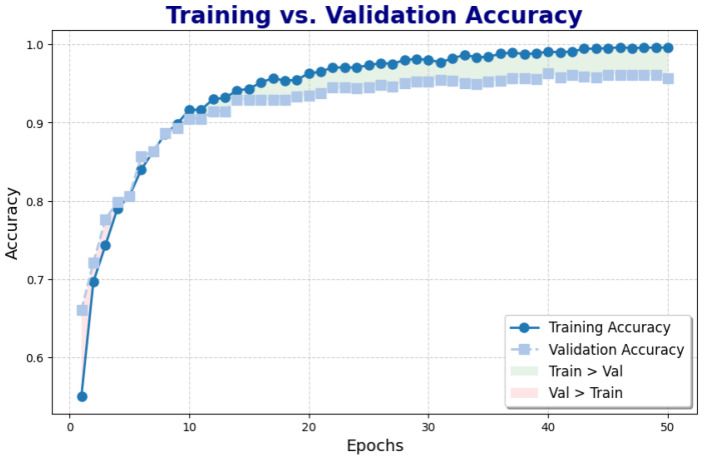
Training vs. validation accuracy throughout training. The graph illustrates periods where training accuracy surpasses validation accuracy (highlighted in light green) and periods where validation accuracy exceeds training accuracy (highlighted in light pink), indicating model behavior across epochs.

Figure 5 shows a graph that illustrates the progression of training and validation accuracy over the course of model training. The blue line represents the accuracy of the training, which initially increases quickly and then plateaus as the model optimally fits the training data. The gray line represents the validation accuracy, which also increases alongside the training accuracy.

The graph is separated into two regions: the green area indicates where the training accuracy exceeds the validation accuracy, suggesting overfitting, as the model performs well on training data but struggles to generalize to validation data. In contrast, the pink area represents the region where the accuracy of the validation exceeds the accuracy of the training, signaling an improved generalization of the training to the validation data.

The key takeaway from this graph is that the model starts by overfitting the training data, but as training progresses, it is able to generalize better to the validation data. The point at which the validation accuracy surpasses the training accuracy, often called the “crossover” point, is typically seen as an optimal stopping point, signaling that the model has achieved a balance between fitting the training data and generalizing to unseen data.

**Figure 6:**
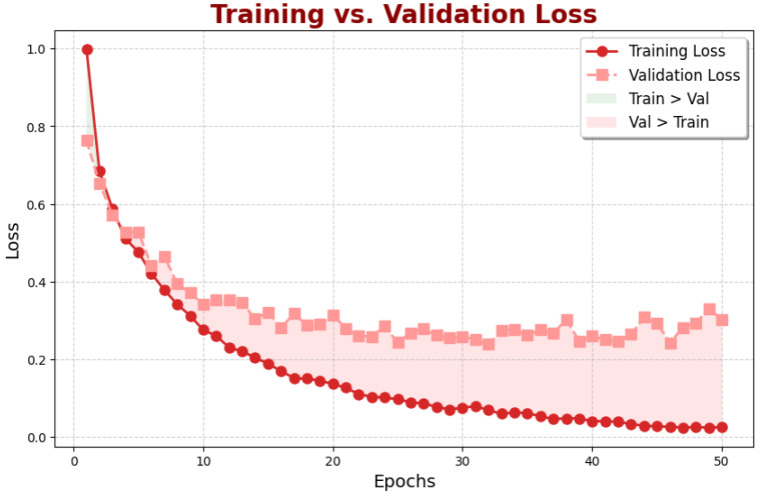
Training vs. validation loss over training epochs. The chart highlights intervals where training loss is lower than validation loss (light pink) and intervals where validation loss is lower than training loss (light green), providing insight into model convergence and generalization performance.

Figure 6 illustrates the progression of training loss and validation during the model’s training process. The red line represents the training loss, initially high but rapidly decreasing as the model adapts to the training data. The pink line shows the validation loss, which also declines, albeit more gradually than the training loss.

This graph illustrates the common challenge of overfitting during the training process. The green area shows the region where the training loss is lower than the validation loss. This indicates that the model overfits the training data and needs to generalize better to the unseen validation data. In contrast, the pink area shows the region where the validation loss is lower than the training loss, suggesting the model has found a better balance between fitting the training data and generalizing to new examples.

### 4.3. Confusion Matrix

**Figure 7:**
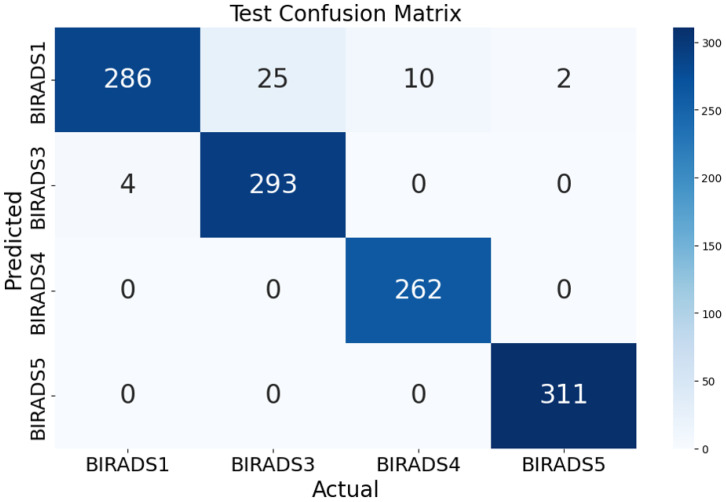
Confusion matrix showing the model’s classification performance across all classes. The matrix provides a detailed view of correctly and incorrectly classified samples, with the diagonal representing true positives and off-diagonal values indicating misclassifications, aiding in assessing model accuracy and error distribution across classes.

Figure 7 shows the test confusion matrix, offering a detailed overview of the classification model performance in various classes. This type of visualization is a commonly used tool in the evaluation of machine learning models.

The rows correspond to the actual classes, and the columns indicate the predicted classes. The values in each cell indicate the number of instances that were classified as the corresponding predicted class when the actual class was the corresponding actual class.

In the classification results, most of the instances were correctly classified in the BIRADS categories. Specifically, BIRADS1 had 286 correct classifications with 4 misclassified as BIRADS3, BIRADS3 had 293 correct classifications with 25 misclassified as BIRADS1, BIRADS4 had 262 correct classifications with 10 misclassified as BIRADS1, and BIRADS5 had 311 correct classifications with 2 misclassified as BIRADS1.

This confusion matrix provides valuable insights into the model’s performance, highlighting its overall accuracy and the specific types of errors it is making.

### 4.4. Probability Distribution Graph

**Figure 8:**
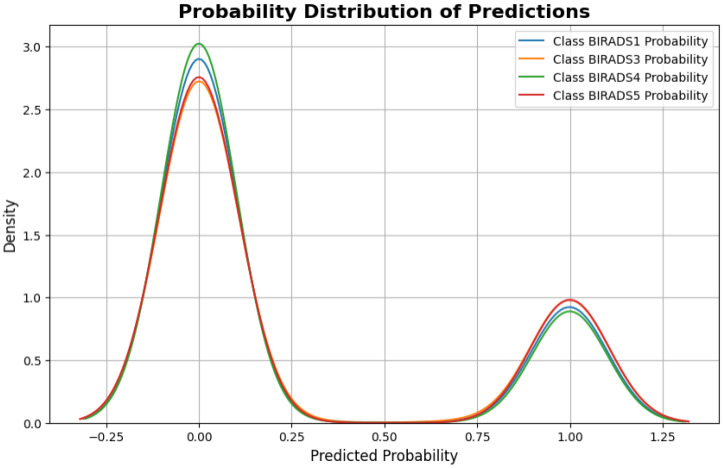
Probability distributions of model predictions for each class, displaying the likelihood assigned to each class across samples. The distribution plots show the spread and concentration of predicted probabilities, highlighting the model’s confidence levels and any overlapping predictions among the four classes.

Figure 8 presents the probability distribution of the predicted probabilities of the model for each class label in the classification task. The *x*-axis represents the predicted probability values, while the y-axis represents the density or probability density function of the predicted probabilities.

The probability distributions for different BIRADS classes reveal the confidence levels of the model across classifications. The BIRADS1 (blue) and BIRADS3 (orange) lines show high probability concentrations, with BIRADS1 showing the highest confidence. The BIRADS4 (green) distribution demonstrates a clear and confident peak, while the BIRADS5 (red) distribution has a slightly broader peak, indicating marginally less certainty in its predictions compared to BIRADS4.

The clear separation and distinct peaks in the probability distributions indicate that the model effectively differentiates between the different BIRADS classes. The relative heights and positions of the peaks indicate the confidence levels of the model for each class prediction.

**Figure 9:**
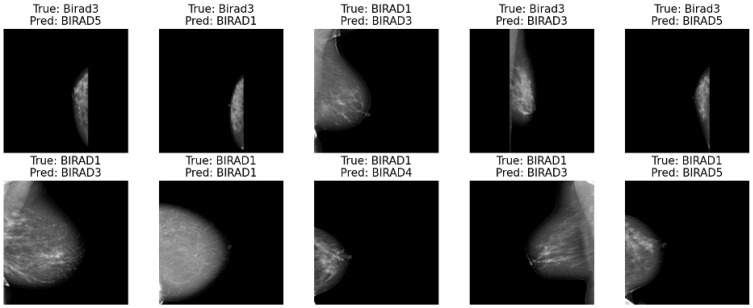
Grid view of model predictions, showcasing ten samples with each image labeled by its true class and predicted class. This visualization includes both correct and incorrect predictions, providing insight into the model’s strengths and challenges in accurately classifying each sample.

Figure 9 displays the classification results of a machine learning model applied to medical images. Each row shows a set of images, where the “True” label represents the actual class of the image, and the “Pred” label represents the class predicted by the model. The key points to highlight in the analysis of these results are as follows:

The model performs well in distinguishing between the different BIRADS classes, as the predicted labels generally match the accurate labels across the examples. There are a few instances where the model has made incorrect predictions, such as predicting BIRADS5 when the actual label is BIRADS3 or predicting BIRADS1 when the accurate label is BIRADS4. These types of misclassifications are essential to analyze further to understand the model’s limitations and areas for improvement.

### 4.5. Comparison with Existing Techniques

A comprehensive comparison with existing state-of-the-art approaches is present in Table 3.

More recently, a 2023 study employing Inception-V3 CNN architecture demonstrated 89.75% accuracy in a four-class classification scenario [35]. The following years saw more sophisticated approaches, with a CNN-SVM hybrid approach proposed in 2022 reached 93.6% accuracy for five classes [36], while another Deep Neural Network (DNN) implementation in 2022 achieving 94.22% accuracy across eight classes [37]. In 2020, researchers implemented Federated Transfer Learning, achieving 84.5% accuracy in a binary classification task [38]. Our proposed MammoViT model, developed in 2024, significantly advances the field by achieving 97.4% accuracy in four-class classification. This superior performance is attributed to our novel approach that combines the power of the ResNet50 architecture with Vision Transformer layers, enhanced by SMOTE balancing for handling class imbalance, and optimized through Keras Tuner for Vision Transformer layer parameters. These results demonstrate a substantial improvement over existing methodologies, establishing a new benchmark in BIRADS classification accuracy.

## 5. Conclusions

The proposed Vision Transformer-based architecture has demonstrated exceptional performance in classifying BIRADS categories, achieving a remarkable 97.4% accuracy. The experimental results, visualized through various performance metrics, strongly support the effectiveness of our approach. Our proposed MammoViT architecture, with its optimized patch embedding process and carefully tuned transformer blocks, proves to be particularly effective in capturing the subtle visual characteristics that differentiate BIRADS categories. The implementation of dynamic patch sizes (2–4 pixels) and optimized projection dimensions (32–64 features) allows the model to process medical imaging data at multiple scales effectively. The incorporation of dropout layers (0.3–0.7) and L1–L2 regularization has successfully addressed potential overfitting issues, as evidenced by the consistent performance across training and validation sets. In conclusion, the experimental results strongly validate our proposed approach, suggesting that this architecture could serve as a valuable tool in supporting medical professionals in breast cancer diagnosis. The high accuracy, combined with robust performance across different BIRADS categories, positions this model as a promising contribution to computer-aided diagnosis systems in medical imaging. While MammoViT achieves high accuracy in BIRADS classification, it has some limitations. The reliance on ResNet-50 may restrict its adaptability to other imaging modalities, and the Vision Transformer increases computational complexity, which could hinder its deployment in resource-constrained environments. Additionally, the use of SMOTE may not fully replicate real-world data distributions. Future work can explore lightweight architectures, alternative balancing techniques, and validation across diverse datasets to enhance generalizability.

## Figures and Tables

**Table 1:** Architectural details of the proposed MammoViT model, incorporating ResNet50 backbone with Vision Transformer layers optimized through Keras Tuner for mammogram classification.

Layer (Type)	Output Shape	Param #	Connected To
input_layer_1 (InputLayer)	(None, 16, 16, 8)	0	-
conv2d (Conv2D)	(None, 4, 4, 64)	8256	input_layer_1[0][0]
reshape (Reshape)	(None, 16, 64)	0	conv2d[0][0]
multi_head_attention (MultiHeadAttention)	(None, 16, 64)	66,368	reshape[0][0], reshape[0][0]
layer_normalization (LayerNormalization)	(None, 16, 64)	128	multi_head_attention[0][0]
dense (Dense)	(None, 16, 128)	8320	layer_normalization[0][0]
dropout_1 (Dropout)	(None, 16, 128)	0	dense[0][0]
dense_1 (Dense)	(None, 16, 64)	8256	dropout_1[0][0]
dropout_2 (Dropout)	(None, 16, 64)	0	dense_1[0][0]
layer_normalization_1 (LayerNormalization)	(None, 16, 64)	128	dropout_2[0][0]
multi_head_attention_1 (MultiHeadAttention)	(None, 16, 64)	66,368	layer_normalization_1[0][0], layer_normalization_1[0][0]
layer_normalization_2 (LayerNormalization)	(None, 16, 64)	128	multi_head_attention_1[0][0]
dense_2 (Dense)	(None, 16, 128)	8320	layer_normalization_2[0][0]
dropout_4 (Dropout)	(None, 16, 128)	0	dense_2[0][0]
dense_3 (Dense)	(None, 16, 64)	8256	dropout_4[0][0]
dropout_5 (Dropout)	(None, 16, 64)	0	dense_3[0][0]
layer_normalization_3 (LayerNormalization)	(None, 16, 64)	128	dropout_5[0][0]
global_average_pooling (GlobalAveragePooling1D)	(None, 64)	0	layer_normalization_3[0][0]
dense_4 (Dense)	(None, 4)	260	global_average_pooling[0][0]

**Table 2:** Classification reports for each class, demonstrating the model’s performance in distinguishing between the four classes.

Class	*P*	*R*	*F*	Support
0	0.99	0.89	0.93	323
1	0.92	0.99	0.95	297
2	0.96	1.00	0.98	262
3	0.99	1.00	1.00	311
*A*			0.97	1193
*M*	0.97	0.97	0.97	1193
*W*	0.97	0.97	0.97	1193

**Table 3:** Comparison with existing state-of-the-art work on the BIRADS dataset.

Reference #	Year	Presented Methodology	Accuracy	Classes
[35]	2023	Inception-V3 CNN	89.75	4
[36]	2022	CNN-SVM	93.6	5
[37]	2022	DNN	94.22	8
[38]	2020	Federated Transfer Learning	84.5	2
Proposed Model	2024	MammoViT	97.4	4

## Data Availability

Datasets can be downloaded from the given link: https://www.kaggle.com/datasets/asmaasaad/king-abdulaziz-university-mammogram-dataset (accessed on 30 October 2024).
